# Gestational Diabetes Is Characterized by Decreased Medium-Chain Acylcarnitines and Elevated Purine Degradation Metabolites across Pregnancy: A Case–Control Time-Course Analysis

**DOI:** 10.1021/acs.jproteome.2c00430

**Published:** 2023-05-02

**Authors:** Hannah Heath, Rodrigo Rosario, Lauren E. McMichael, Rob Fanter, Noemi Alarcon, Adilene Quintana-Diaz, Kari Pilolla, Andrew Schaffner, Elissa Jelalian, Rena R. Wing, Alex Brito, Suzanne Phelan, Michael R. La Frano

**Affiliations:** † Department of Food Science and Nutrition, California Polytechnic State University, San Luis Obispo, California 93407, United States; ‡ College of Agriculture, Food and Environmental Sciences, California Polytechnic State University, San Luis Obispo, California 93407, United States; § Cal Poly Metabolomics Service Center, California Polytechnic State University, San Luis Obispo, California 93407, United States; ∥ Department of Kinesiology and Public Health, California Polytechnic State University, San Luis Obispo, California 93407, United States; ⊥ Center for Health Research, California Polytechnic State University, San Luis Obispo, California 93407, United States; # Department of Statistics, California Polytechnic State University, San Luis Obispo, California 93407, United States; ∇ Department of Psychiatry and Human Behavior, Warren Alpert Medical School at Brown University, Providence, Rhode Island 02903, United States; ○ Laboratory of Pharmacokinetics and Metabolomic Analysis. Institute of Translational Medicine and Biotechnology. I.M. Sechenov First Moscow State Medical University, 119991 Moscow, Russia; ◆ World-Class Research Center “Digital Biodesign and Personalized Healthcare”, I.M. Sechenov First Moscow State Medical University, 119991 Moscow, Russia

**Keywords:** gestational diabetes mellitus, acylcarnitines, purine degradation pathway, fatty acid oxidation, metabolomics, omics

## Abstract

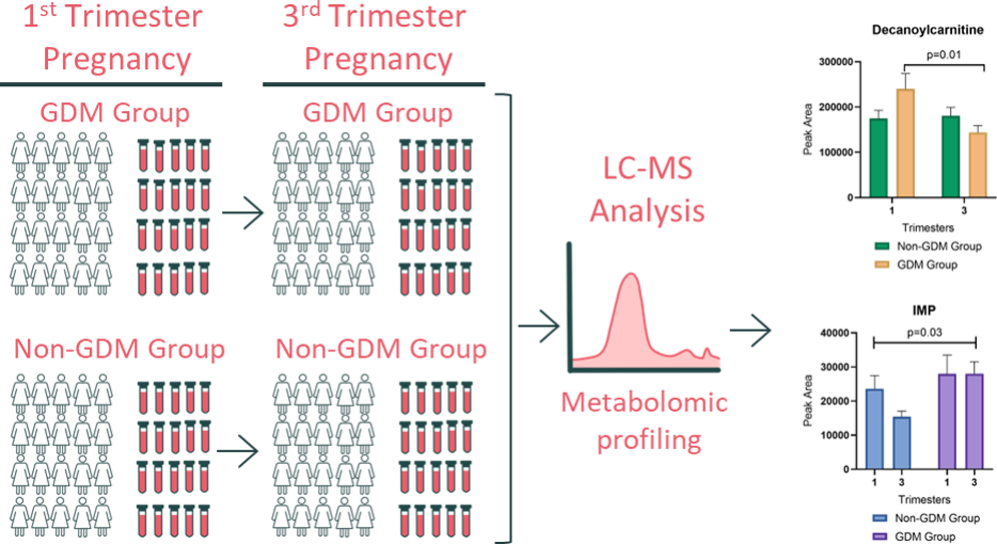

Gestational Diabetes Mellitus (GDM) results in complications affecting both mothers and their offspring. Metabolomic analysis across pregnancy provides an opportunity to better understand GDM pathophysiology. The objective was to conduct a metabolomics analysis of first and third trimester plasma samples to identify metabolic differences associated with GDM development. Forty pregnant women with overweight/obesity from a multisite clinical trial of a lifestyle intervention were included. Participants who developed GDM (*n* = 20; GDM group) were matched with those who did not develop GDM (*n* = 20; Non-GDM group). Plasma samples collected at the first (10–16 weeks) and third (28–35 weeks) trimesters were analyzed with ultra-performance liquid chromatography–mass spectrometry (UPLC-MS). Cardiometabolic risk markers, dietary recalls, and physical activity metrics were also assessed. Four medium-chain acylcarnitines, lauroyl-, octanoyl-, decanoyl-, and decenoylcarnitine, significantly differed over the course of pregnancy in the GDM vs Non-GDM group in a group-by-time interaction (*p* < 0.05). Hypoxanthine and inosine monophosphate were elevated in the GDM group (*p* < 0.04). In both groups over time, bile acids and sorbitol increased while numerous acylcarnitines and α-hydroxybutyrate decreased (*p* < 0.05). Metabolites involved in fatty acid oxidation and purine degradation were altered across the first and third trimesters of GDM-affected pregnancies, providing insight into metabolites and metabolic pathways altered with GDM development.

## Introduction

Gestational diabetes mellitus (GDM) is defined as diabetes diagnosed during pregnancy that is not clearly overt diabetes, which affects approximately 10–15% of pregnancies in the United States each year.^1^ Economic costs of GDM are estimated to be up to $1.6 billion per year.^2^ While information regarding the etiology of GDM remains unclear, various risk factors have been suggested, including obesity during pregnancy, polycystic ovarian syndrome (PCOS), and potential genetic predisposition. Diet and physical activity can also contribute to GDM development.^3^ Screening and diagnosis of GDM consist of two well-established methods: the one-step approach derived from the International Association of Diabetes and Pregnancy Study Groups (IADPSG) criteria and the two-step approach derived from Carpenter and Coustan’s interpretation of O’Sullivan’s criteria.^1^ GDM may lead to complications that can affect either mother, fetus, or child development. Mothers with GDM may develop hypertension and preeclampsia, while their offsprings may experience macrosomia-related complications and increased risk of obesity, later GDM development, and motor and developmental disorders.^3−5^

Biological markers, or “biomarkers,” refer to measurable objective indicators relative to a specific biological state or condition.^6^ While the use of biomarkers in both basic and clinical research has increased, biomarkers predictive of GDM are lacking.^7,8^ Metabolomics, the extensive study of metabolites in biological samples, has been vital for propelling and strengthening such biomarker investigations. In metabolomics studies, biomarker discovery revolves around the profiling of metabolites in biofluids, cells, and tissues.^9^ The discovery of potential metabolomic biomarkers for GDM may provide insight into its etiology and pathophysiology, in turn leading to advances in the prediction, prevention, diagnosis, and treatment of GDM.^10^ A previous first trimester GDM study conducted by our lab identified fatty acid utilization and purine degradation products, as well as kynurenic acid and tricarboxylic acid cycle intermediates, to be altered in early pregnancy.^11^

More research is required to investigate the metabolic changes associated with gestational diabetes development to confirm previous findings and to further understand the disease etiology. The objective of this present study was to conduct metabolomics analysis of first and third trimester plasma samples to identify metabolic differences associated with GDM development.

## Participants and Methods

This secondary analysis used samples from the Healthy Beginnings/Comienzo Saludables studio, a randomized controlled clinical trial (RCT) focused on the outcomes of behavior lifestyle change on weight gain during gestation that is part of the Lifestyle Interventions for Expectant Moms (LIFE-Moms) consortium.^12^ All procedures were approved by the institutional review boards (IRBs) of the participating institutions. The trial was registered at www.clinicaltrials.gov as NCT01545934. This two-site trial included participants at the following study sites: Miriam Hospital with Women and Infants Hospitals in Providence, Rhode Island, and California Polytechnic State University, San Luis Obispo, CA. Participants were randomly assigned within site and to two different intervention groups: a control group that received standard care and a treatment group that received a multicomponent lifestyle intervention that consisted of diet, exercise, and behavioral changes. Since the treatment group had no effect on GDM occurrence (*p*-value = 0.7),^12^ samples from both groups were included in the secondary analysis. Third trimester data consisted of a total of 20 GDM cases and 20 healthy controls collected from the Rhode Island (*n* = 33) and California (*n* = 17) sites. Participants with GDM were matched on age, BMI, ethnicity, and treatment group with those who did not develop GDM. This study was performed in accordance with the Declaration of Helsinki. Informed consent was obtained from all participants included in this study.

Fasting plasma samples were collected prior to GDM diagnosis during the first trimester (10–16 weeks of gestation) and after GDM diagnosis during the third trimester (28–35 weeks of gestation). Between 24 and 27 weeks of gestation, research staff confirmed gestational diabetes via a 2 h 75 g oral glucose tolerance test (OGTT) using the IADPSG criteria. In circumstances in which a study-measured OGTT was not obtained, charts were abstracted for the confirmation of GDM. The clinical measures used varied across clinics, including the use of a 1 h 50 g value ≥200 mg/dL, a clinical chart indication of “diabetes,” or confirmation of GDM by the American College of Obstetricians and Gynecologists-endorsed, 2-step approach.^13^

### Metabolomics Analysis

Samples were randomized and assigned new IDs prior to processing and analysis. Targeted metabolomics assays for primary metabolomics, biogenic amines, and lipidomics were used to analyze plasma samples using ultra-performance liquid chromatography–tandem quadrupole mass spectrometry (UPLC-MS) using modified published methods.^14^ Specific precursor and product ions were screened for hundreds of metabolites from several metabolite classes, and semi-quantitative data (peak area) was produced. More specifically, the primary metabolomics assay screens for primary metabolites such as carbohydrates, carboxylic acids, purines/pyrimidines/nucleotides/nucleosides, amino acid derivatives, sterols, and vitamins, in addition to other compounds. The biogenic amine assay screens for amine compounds, including amino acids, amino acid derivatives, purines/pyrimidines/nucleotides/nucleosides, quaternary ammonium compounds, acylcarnitines, and imidazoles. The lipidomics assay detects complex lipids such as phospholipids, lysophospholipids, and sphingomyelins.

Briefly, 25 μL of plasma was added to 1.5 mL tubes before addition of 10 μL of 1 μM internal standard solution, followed by 750 μL of chilled methanol. Subsequently, samples were vortexed for 30 s prior to centrifugation at 15,000*g* for 10 min. The same volume of supernatant per sample was moved to 1.5 mL high-performance liquid chromatography (HPLC) amber glass vials, dried by centrifugal vacuum evaporation, and reconstituted in a solution containing 100 μL a 3:1 acetonitrile/methanol containing internal standard 1-cyclohexyl-ureido, 3-dodecanoic acid (CUDA; Sigma-Aldrich, St. Louis, MO) solution. The reconstituted solution was then vortexed for 30 s and put on ice for 10 min. The solution was centrifuged at 10,000*g* for 3 min in microfilter tubes and then transferred to HPLC vials for UPLC-MS analysis.

UPLC-MS analyses were performed on a Waters Acquity I-Class UPLC (Waters, Milford, MA) coupled with an API 4000 QTrap (Sciex, Framingham, MA) using multiple reaction monitoring (MRM). Peak areas were quantified with AB Sciex MultiQuant version 3.0. Primary metabolomics and biogenic amines utilized multiple reaction monitoring (MRM) that has been previously published.^14^ The lipidomics assay used full scan MS over *m*/*z* 400–1000 utilizing Q1 scans at unit mass resolution, with specific lipid species being identified using a range to capture the full width of the monoisotopic ion, as described previously.^14^ For the primary metabolomics assay, metabolites were separated with a 150 × 2.0 mm Luna NH2 column (Phenomenex, Torrance, CA) and detected using negative ion mode electrospray ionization. For the biogenic amines assay, metabolites were separated using a 150 × 2.1 mm Atlantis HILIC column (Waters) and detected using positive ion mode electrospray ionization. For the lipidomics assay, metabolites were separated with a 150 × 3.0 mm Prosphere HP C4 column (Grace, Columbia, MD) and detected using positive ion mode electrospray ionization.

Primary metabolomics and biogenic amine assay metabolite identities were confirmed using pure standards to establish retention time and MRM, as well as to optimize instrument parameters. Standards included those from the Mass Spectrometry Metabolite Library of Standards (MSMLS; Sigma-Aldrich), as well as individually purchased standards from Sigma-Aldrich, Cambridge Isotope Laboratories, Inc. (Tewksbury, MA), and Cerilliant Corporation (Round Rock, TX). For the lipidomics assay, the SPLASH LIPIDOMIX Mass Spec Standards purchased from Avanti Polar Lipids Inc. (Alabaster, AL) were used to identify the retention time of select lipid species to establish retention time indexes that adjust for retention time differences in specific lipid species between our method and those provided by Townsend et al.^14^ To confirm approximate retention time ranges for phosphatidylcholines, phosphatidylethanolamines, lysophosphatidylcholines, lysophosphatidylethanolamines, and sphingomyelins, purified egg yolk extracts (99–97% purity by TLC; Sigma-Aldrich) of each targeted lipid class, consisting of a variety of species, were analyzed. Surrogate standards used in the primary metabolomics assay included succinate-^13^C_4_, sorbitol-1,1,2,3,4,5,6,6-d_8_, octanoate-^13^C_8_, adenine-2-d_1_, and histamine-α,α,β,β-d_4_, while the biogenic amine assay used L-tryptophan-^13^C_11_, adenine-2-d_1_, 2-(3,4-dihydroxyphenyl)ethyl-1,1,2,2-d_4_-amine, and histamine-α,α,β,β-d_4_. These standards were used to monitor extraction efficiency and recovery percentage for each sample analyzed. Surrogates were purchased from Santa Cruz Biotechnology, Inc. (Dallas, TX), CDN Isotopes Inc. (Pointe-Claire, Quebec, Canada), and Cambridge Isotope Laboratories, Inc. To control for instrument and injection parameters, the internal standard CUDA (Sigma-Aldrich), included in the reconstitution solvent that was added post-extraction, was utilized. All primary metabolomics and biogenic amine raw data were normalized to CUDA (Sigma-Aldrich), and the lipidomics assay raw data were normalized to the mTIC.

During data processing, compounds were excluded from the data set if their signal-to-noise ratio was less than 3:1 or if their background (as determined by method blank response) was greater than 50% of the average sample response. Five replicates of the current study samples were separately extracted and analyzed to assess reproducibility. A pooled plasma sample collected from a different study was used as a long-term reference QC sample for an intra- and inter-study assessment of data. All samples were run in a single batch.

### Diet and Physical Activity Analyses

Measurements of diet and physical activity were taken to assess their potential impact on metabolite alterations. Dietary intake was assessed using the National Cancer Institute Automated Self-Administered 24 h Recall during two random days of the week at study entry and then again at 35 weeks. Dietary data collected included total daily calories, macronutrient, and micronutrient intake estimations.^15^ Physical activity was measured via an accelerometer worn for an average of 6 days at study entry and 35 weeks to measure total minutes spent participating in varying degrees of activity intensities.^12^ Additional activity was estimated via a physical activity questionnaire completed at study entry and at 35 weeks of pregnancy.

### Statistical Analyses

A general linear model (GLM) was used with random effects for individuals and fixed effects for case/control, time, and case/control by time interaction. Additional fixed effects were ethnicity, treatment group, age, BMI, and BMI by time interaction. The statistical software used was JMP Pro 16.0.0. Multiple comparisons were accounted for with the Benjamini–Hochberg procedure with an experiment-wise error rate of 5% to adjust the reported *p*-values.^16^ Prior to multivariate analysis, a linear model was fitted using R version 3.6.3 to adjust for differences in metabolite values associated with BMI, ethnicity, and RCT treatment group; the residuals from this model were subsequently used for multivariate analysis, including partial least squares discriminant analysis (PLS-DA) in MetaboAnalyst.^17^

## Results

The GDM vs non-GDM groups did not significantly differ in age, BMI, or ethnicity (Table 1). Group-by-time analysis of the cardiometabolic risk marker data showed no statistically significant differences between groups over time (Table 2). A group-by-time interaction showed decreased carbohydrate (grams) and α-carotene intake in the GDM compared to the non-GDM group (Supporting Information Table S1). The estimated dietary intake of energy, percentages of macronutrients consumed, total protein and fat, and micronutrients were not different between the GDM and the non-GDM group over time. Group-by-time analysis of physical activity data found sedentary activities to increase in the GDM group over time, while walking pace was found to decrease in the non-GDM group over time (Supporting Information Table S2).

**Table 1:** Participant Characteristics for Non-GDM and GDM Groups^a^

characteristics	non-GDM (*n* = 20)	GDM (*n* = 20)	*P*-value
age	31 ± 5.8^b^	31.9 ± 5.2	0.61
BMI	32.3 ± 5.1	34.1 ± 6.0	0.33
weight at 35 weeks in kilograms	92.0 ± 12.8	98.3 ± 19.3	0.07
hispanic			
no (%)	11 (27.5)	13 (32.5)	
yes (%)	9 (22.5)	7 (17.5)	
BMI classification			
obese (%)	18 (45)	18 (45)	
overweight (%)	2 (5)	2 (5)	
annual household income			
> = $49k annual household income (%)	10 (25)	12 (30)	
> = $50k annual household income (%)	10 (25)	8 (20)	
education			
high school or less (%)	5 (12.5)	5 (12.5)	
postgraduate work (%)	5 (12.5)	2 (5)	
some college or college degree (%)	10 (25)	13 (32.5)	
marital status			
married or living with significant other (%)	19 (47.5)	17 (42.5)	
not married/separated/divorced/widowed (%)	1 (2.5)	3 (7.5)	
parity			
multiparous (%)	13 (32.5)	16 (40)	
primiparous (%)	7 (17.5)	4 (10)	

a Abbreviation*:* BMI, body mass index; Non-GDM, nongestational diabetes mellitus; GDM, gestational diabetes mellitus; SD, standard deviation.

b Mean ± SD (all such values).

**Table 2:** Cardiometabolic Risk Markers for Non-GDM and GDM Group-by-Time Comparisons and Test of Interaction^a^

		Non-GDM		GDM		
metabolite	trimester	mean	SD	mean	SD	*P*-value
glucose (mg/dL)	1st	90.3	8.2	91.9	8.8	0.59
	3rd	85.6	7.0	85.3	8.31	
insulin (μU/mL)	1st	18.9	16.0	21.0	10.9	0.72
	3rd	20.3	10.6	22.5	9.3	
HOMA-IR	1st	4.6	4.4	4.9	2.8	0.63
	3rd	4.5	2.5	4.8	2.2	
HDL cholesterol (mg/dL)^c^	1st	65.3	14.6	57.3	12.4	0.63
	3rd	65.3	18.2	58.8	17.5	
LDL cholesterol^b^	1st	89.1	26.8	89.8	32.9	0.14
	3rd	121.8	38.3	106.5	35.1	
total cholesterol (mg/dL)	1st	181.0	32.4	176.8	35.9	0.15
	3rd	234.5	42.9	211.6	38.4	
leptin (μg/L)	1st	54.2	15.1	52.6	14.2	0.59
	3rd	61.2	12.8	63.8	26.5	
triglycerides (mg/dL)	1st	133.0	63.2	148.5	61.5	0.14
	3rd	244.6	85.5	240.7	92.8	
C-peptide (ng/mL)	1st	2.5	1.4	2.9	1.0	0.20
	3rd	3.6	1.1	3.8	1.6	

a Abbreviation*:* Non-GDM, nongestational diabetes mellitus; GDM, gestational diabetes mellitus; SD, standard deviation; HOMA-IR, homeostatic model assessment for insulin resistance; HDL, high-density lipoprotein; LDL, low-density lipoprotein.

b Friedewald LDL cholesterol.

c HDL chol, direct.

Metabolomics analysis detected 131 metabolites. The full list of metabolites is shown in Supporting Information Table S3. A significant group-by-time interaction found four medium-chain acylcarnitines, lauroyl-, octanoyl-, decanoyl-, and decanoylcarnitine to decrease over the course of pregnancy in the GDM group but not in the non-GDM group (*p* < 0.05) (Table 3). Higher levels of purine degradation metabolites inosine monophosphate (IMP) and hypoxanthine were observed in the GDM group compared to the non-GDM group (*p* < 0.04) (Table 4). Additionally, numerous metabolite alterations were seen in both the GDM and the non-GDM groups over time: There was a significant decrease in the short-chain acylcarnitine acetylcarnitine, the medium-chain acylcarnitines lauroyl-, dodecanoyl-, propionyl-, decenoyl-, decanoyl-, and octanoylcarnitine, and long-chain acylcarnitines palmitoyl-, 3-hydroxy-palmitoleoyl-, and linoleylcarnitine (*p* < 0.04). The bile acids taurocholate and glycocholate increased in both groups over time (*p* < 0.05), while the bile acid SUM-taurodeoxycholate/taurochenodeoxycholate decreased in both groups over time (*p* < 0.05). Adenosine diphosphate (ADP) and uracil, both metabolites of the purine degradation pathway, decreased in both groups over time (*p* < 0.03), as did the lipids phosphatidylcholine (PC) C38:6 and lysophosphatidylcholine (LPC) C20:4 (*p* < 0.05). Additionally, sorbitol and threonine increased in both groups over time, while α-hydroxybutyrate decreased (*p* < 0.05) (Table 5). Taurocholate, glycocholate, ADP, sorbitol, and lauroylcarnitine were the only metabolites that passed the false discovery rate (FDR). PLS-DA multivariate analysis did not distinguish groups (Supporting Information Figures S1 and S2).

**Table 3:** Group-by-Time Metabolomics Comparison and Test of Interaction^a^,^b^

		non-GDM		GDM		
metabolite	trimester	mean	SD	mean	SD	*P*-value
decanoylcarnitine	1st	174,549	81,051	239,942	151,319	0.01
	3rd	180,616	82,273	143,849	67,053	
lauroylcarnitine	1st	119,163	38,510	159,880	110,840	0.03
	3rd	102,701	50,987	77,695	33,143	
octanoylcarnitine	1st	42,204	21,904	52,096	28,164	0.03
	3rd	41,891	20,885	32,361	15,556	
decenoylcarnitine	1st	79,466	35,919	103,554	36,903	0.04
	3rd	71,171	22,962	70,889	29,575	

a Mean and SD data are in the peak area.

b Abbreviation: Non-GDM, nongestational diabetes mellitus; GDM, gestational diabetes mellitus; SD, standard deviation.

**Table 4:** Group Effect Metabolomics Results^a^^b^

		non-GDM		GDM		
metabolite	trimester	mean	SD	mean	SD	*P*-value
IMP	1st	23,602	17,572	27,986	24,521	0.03
	3rd	15,404	7,455	28,049	23,602	
hypoxanthine	1st	51,048	36,782	66,789	57,744	0.03
	3rd	35,725	15,704	60,907	51,048	

a Mean and SD data are in peak area.

b Abbreviation: IMP, inosine monophosphate; Non-GDM, nongestational diabetes mellitus; GDM, gestational diabetes mellitus; SD, standard deviation.

**Table 5:** Time Effect Metabolomics Results^a^,^b^

		non-GDM		GDM		
metabolite	trimester	mean	SD	mean	SD	*P*-value
ADP	1st	34,974	13,217	36,974	12,209	1.2 × 10^–7^
	3rd	20,406	9,289	22,222	8,199	
taurocholate	1st	19,672	14,684	26,156	17,595	2.1 × 10^–3^
	3rd	54,390	41,559	135,638	238,470	
lauroylcarnitine	1st	119,163	38,510	15,988	110,840	1.6 × 10^–4^
	3rd	102,701	50,987	77,695	33,143	
acetylcarnitine	1st	11,435,585	5,064,398	11,273,382	6,169,041	2.1 × 10^–3^
	3rd	9,710,870	5,216,248	9,182,298	3,575,290	
dodecenoylcarnitine	1st	190,292	86,236	215,326	110,045	2.8 × 10^–3^
	3rd	173,245	97,347	129,276	54,191	
propionylcarnitine	1st	726,775	497,522	650,585	549,549	2.9 × 10^–3^
	3rd	455,354	410,046	580,343	512,110	
sorbitol	1st	454,844	381,746	443,932	378,183	3.5 × 10^–3^
	3rd	594,539	757,973	517,314	193,581	
decenoylcarnitine	1st	79,466	35,919	103,554	36,903	3.6 × 10^–3^
	3rd	71,171	22,962	70,889	29,575	
glycocholate	1st	83,360	66,174	101,830	114,122	4.3 × 10^–3^
	3rd	150,150	151,037	261,660	252,458	
decanoylcarnitine	1st	174,549	81,051	239,942	151,319	0.02
	3rd	180,616	82,273	143,849	67,053	
palmitoylcarnitine	1st	395,516	129,575	527,613	226,574	0.02
	3rd	333,923	136,424	492,060	530,447	
threonine	1st	346,556	217,080	282,642	185,456	0.02
	3rd	420,224	228,370	401,627	187,751	
3-OH-palmitoleoylcarnitine	1st	16,822	10,067	22,468	14,537	0.02
	3rd	13,413	6,800	17,492	14,995	
uracil	1st	296,204	114,790	327,570	113,495	0.02
	3rd	264,386	92,718	229,216	76,769	
LPC C20:4	1st	35,677,929	13,870,554	38,098,481	7,898,445	0.03
	3rd	29,964,657	11,112,917	34,013,116	10,639,903	
PC C38:6	1st	357,450,000	97,842,771	356,000,000	64,623,607	0.03
	3rd	326,500,000	78,966,015	328,800,000	74,771,652	
octanoylcarnitine	1st	42,204	2,1904	52,096	28,164	0.03
	3rd	41,891	20,885	32,361	15,556	
α-hydroxybutyrate	1st	1,589,962	574,001	2,058,695	918,528	0.03
	3rd	1,383,531	384,638	1,397,649	578,636	
linoleylcarnitine	1st	223,197	97,106	284,132	168,016	0.04
	3rd	175,940	83,017	253,539	213,126	
SUM_Taurodeoxycholate_taurochenodeoxycholate	1st	18,258	16,893	17,923	16,183	0.04
	3rd	12,033	6,081	13,811	8,975	

a Mean and SD data are in peak area.

b Abbreviation: ADP, adenosine diphosphate; LPC, lysophosphatidylcholine; PC, phosphatidylcholine; Non-GDM, nongestational diabetes mellitus; GDM, gestational diabetes mellitus; SD, standard deviation.

## Discussion

This study utilized metabolomics to highlight metabolites altered across the first and third trimesters in GDM vs non-GDM groups. Medium-chain acylcarnitines were observed to decrease in the GDM group over time, while purine degradation metabolites were elevated in the GDM group compared to the non-GDM group. Metabolites altered in the GDM group were primarily related to fatty acid oxidation, inflammation, and insulin resistance.

### Metabolites Altered in the GDM Group over Time

**Figure 1:**
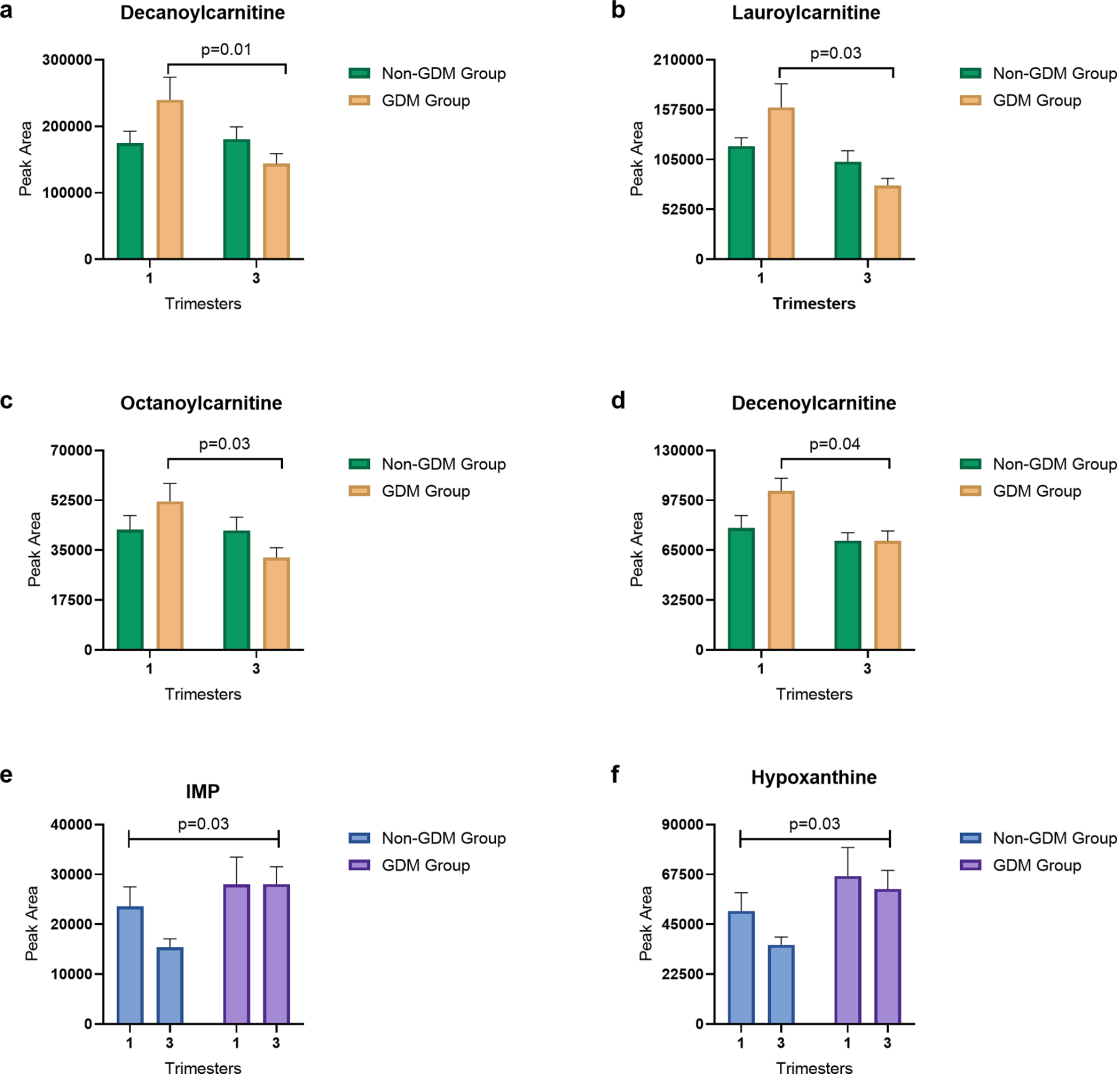
Bar charts of metabolites with a significant group-by-time interaction, including (a) decanoylcarnitine, (b) lauroylcarnitine, (c) octanoylcarnitine, and (d) decenoylcarnitine. Metabolites with a significant group effect shown include (e) hypoxanthine and (f) inosine monophosphate (IMP).

Decanoyl-, decenoyl-, dodecanoyl-, and lauroylcarnitine, all of which are medium-chain acylcarnitines, decreased over the course of pregnancy in the GDM group vs the non-GDM group (Figure 1a–d). A prediagnosis GDM study found both decanoyl- and dodecanoylcarnitine to decrease in the plasma samples of women with GDM,^18^ while a third trimester study found decanoylcarnitine to increase.^19^ One longitudinal study found medium-chain acylcarnitine C8:1 to decrease between the second and third trimesters in the GDM group compared to the non-GDM group.^20^ Additionally, elevated medium-chain acylcarnitines have also been observed among people with T2D.^21,22^

**Figure 2:**
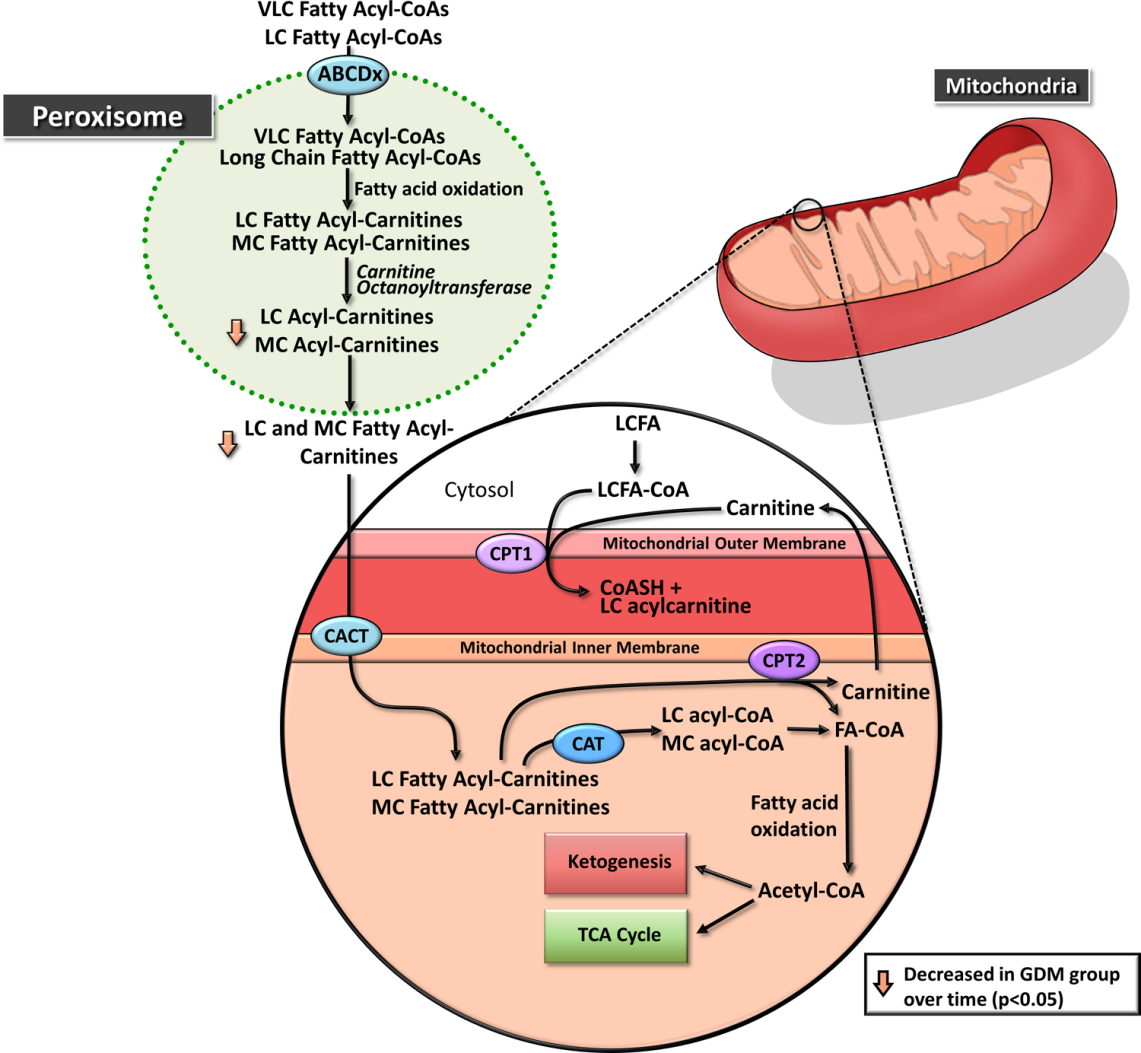
Proposed hypothetical fatty acid oxidation pathway related to metabolites altered in group-by-time interaction among women who develop gestational diabetes mellitus (GDM). ABCDx: members of the peroxisomal ABC transporter family; CPT1: carnitine palmitoyltransferase 1; CACT: carnitine–acylcarnitine translocase; CPT2: carnitine palmitoyltransferase 2; CAT: carnitine acetyltransferase; FA-CoA: fatty acid-CoA; LCFA: long-chain fatty acid; LC acyl-carnitine: long-chain acyl-carnitine; LC-acyl-CoA: long-chain acyl-CoA; MC acyl-CoA: medium-chain acyl-CoA; MC acyl-carnitine: medium-chain acyl-carnitine; TCA Cycle: tricarboxylic acid cycle; VLC-acyl-CoA: very long-chain acyl-CoA.

As noted in Schooneman et al. review of acylcarnitines and insulin resistance, insulin resistance may be accompanied by excessive fatty acid oxidation (FAO) combined with a depletion of TCA intermediates, which may overload the mitochondria and result in incomplete FAO as reflected by increases in plasma acylcarnitines.^21^ As such, the decline in medium-chain acylcarnitines from the first to the third trimester in the GDM group appears to conflict with this mechanism, as insulin resistance increases over the course of pregnancy and thus would be expected to result in an increase in acylcarnitines. Though there are several factors that may have influenced this alteration, the mechanism behind this unexpected decrease in plasma acylcarnitines remains unclear. Dietary analysis indicated decreased carbohydrate intake in the third trimester in the GDM vs the non-GDM group. Because participants were diagnosed with GDM several weeks prior to the third trimester sample collection, this resulting dietary change may have altered acylcarnitine processing. Another possibility for this decrease in acylcarnitines may relate to chain length, as acylcarnitines are processed differently depending on their chain ength. Carnitine octanoyltransferase (CrOT), a peroxisomal enzyme, has a high affinity for medium-chain fatty acids and is important for the processing of medium-chain acylcarnitines by transesterifying medium-chain acyls into medium-chain acylcarnitines^23^ (Figure 2). The dysregulated energy metabolism occurring in GDM may alter CrOT activity, thus decreasing plasma medium-chain acylcarnitine levels. However, we were unable to investigate CrOT activity in this study, limiting our ability to investigate this potential mechanism., Research focusing on medium-chain acylcarnitines in third trimester GDM is lacking, and follow-up studies are necessary to fully understand the observed decrease in these metabolites.

### Metabolites Elevated in the GDM vs Non-GDM Group

IMP and hypoxanthine, both metabolites involved in the purine degradation pathway, were higher in the GDM group compared to the non-GDM group (Figure 1e,f). Our previous study in first trimester samples revealed elevated plasma hypoxanthine with GDM,^11^ an observation also made in Zhao et al. study of early pregnancy GDM markers. In a longitudinal study by Law et al., hypoxanthine was found to be elevated in the urinary samples of women with GDM during all trimesters.^24^ Uric acid, a downstream product of hypoxanthine oxidation, has also been elevated in GDM patients.^18^ Other GDM studies have not reported changes in IMP.

**Figure 3:**
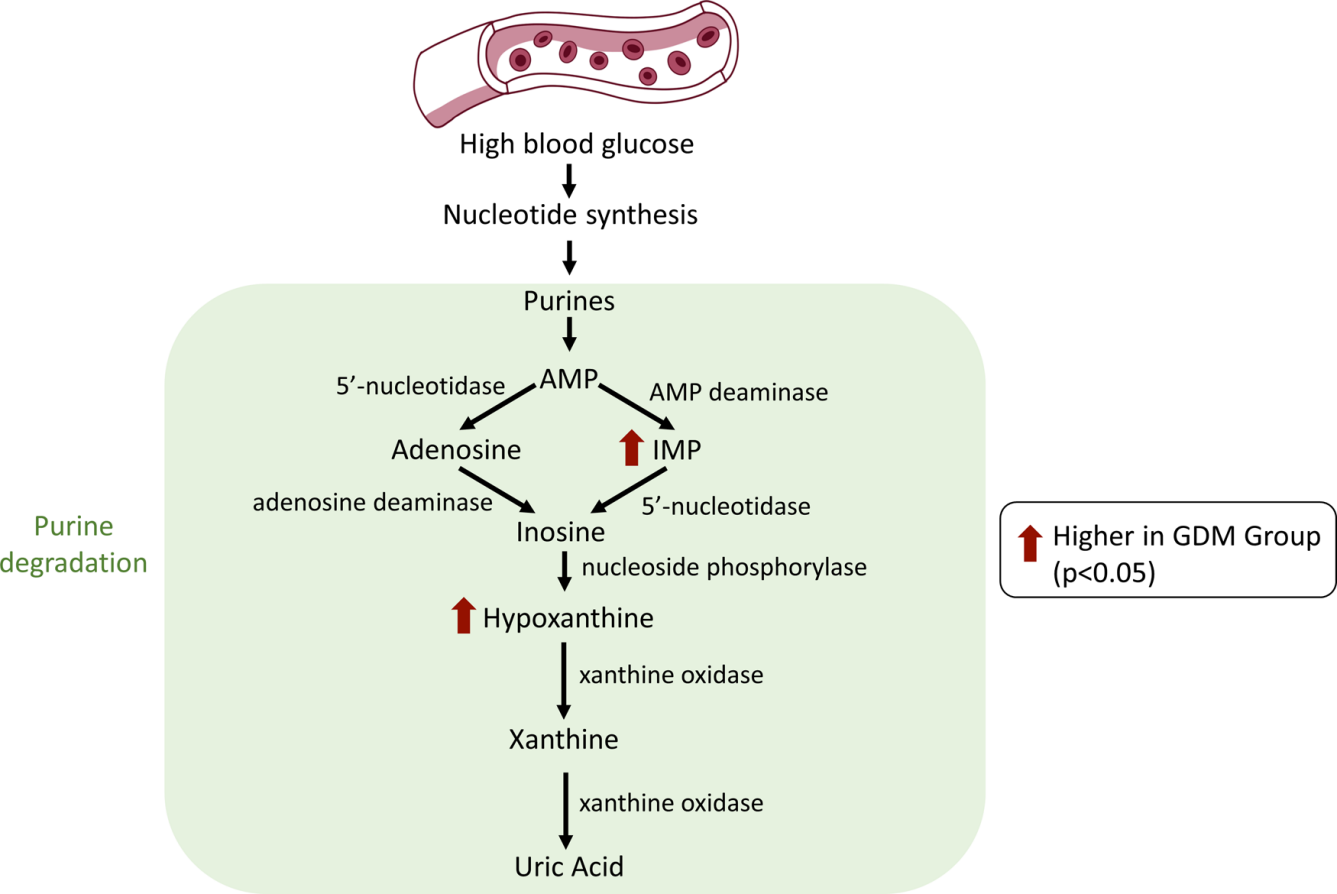
Proposed hypothetical purine degradation pathway related to metabolites altered in group effects altered among women who develop gestational diabetes mellitus (GDM). AMP: adenosine monophosphate; IMP: inosine monophosphate.

A study of erythrocyte levels of purine nucleotides and metabolites in patients with T1D and T2D found a significant increase in nucleotide synthesis in comparison with healthy controls, as well as an increase in the purine degradation metabolites IMP and hypoxanthine. This indicates an increase in nucleotide synthesis with hyperglycemia and, subsequently, an increase in purine degradation metabolites^25^ (Figure 3). Similarly, elevated uric acid is commonly seen in diabetes, further highlighting the association between purine degradation and insulin resistance (Sharaf El Din, 2017). As noted in Law et al. review of the pathophysiology and pathogenesis of GDM, the xanthine oxidase-driven process of oxidizing hypoxanthine into xanthine and then uric acid generates superoxide anions which are associated with increased inflammation and impaired insulin secretion.^26^ Therefore, the altered purine degradation pathway observed in this study may be due to the increase in nucleotide synthesis seen with glucose dysregulation, thus resulting in an increase in ROS, which contributes to insulin resistance. However, further research is needed to discern whether altered purine metabolites influence insulin resistance development through this pathway or whether changes in these metabolites are instead a reflection of said insulin resistance.

### Metabolites Altered in Both Groups over Time

In both the GDM and the non-GDM groups, over time, there was a significant decrease in acylcarnitine of all lengths. This is consistent with previous longitudinal studies that have found acylcarnitines to decrease over the course of both healthy and GDM-affected pregnancies.^20,27−29^

Several reasons for this decrease in acylcarnitines have been proposed. The drop in acylcarnitines levels across pregnancy may be a result of the elevated demands of the fetus, resulting in higher placental transfer.^29^ Additionally, several studies have found elevated urinary excretion of acylcarnitines during late pregnancy, potentially resulting in lower plasma acylcarnitines over time.^27,30^ The reason behind this elevated urinary excretion is not fully understood, but it may be related to carnitine’s function as a facilitator of the removal of excess and potentially toxic acyl groups from cells, resulting in the excretion of these acyl groups as acylcarnitines in the urine.^27^

α-hydroxybutyrate decreased in both groups over the course of pregnancy. α-hydroxybutyrate appears to be a marker of insulin resistance and dysfunctional glucose metabolism^31^ and has previously been found to be higher in first trimester GDM pregnancies,^11^ as well as during the second and third trimesters of GDM.^32^ α-hydroxybutyrate is a breakdown product of α-ketobutyrate, which can be derived from both threonine catabolism and glutathione anabolism.^31^ Interestingly, threonine was observed to increase over the course of pregnancy in both groups, which agrees with several studies that have found this amino acid to be elevated in the third trimester compared to the first and second trimesters of healthy pregnancies.^28,29,33^ This may indicate decreased threonine catabolism in the third trimester, which could explain the decrease in α-hydroxybutyrate. Additionally, these third trimester samples were collected several weeks after GDM diagnosis, and therefore it is likely that participants initiated dietary and lifestyle changes, as evidenced by the observed decrease in carbohydrate intake and the decrease in blood glucose levels over time. This could be another contributor to the decrease in α-hydroxybutyrate over time, as these changes may have improved insulin resistance and glucose metabolism.

Purine degradation metabolites ADP and uracil were found to decrease in both groups over time. As mentioned previously, purine degradation metabolites have been found to increase with hyperglycemia.^25^ Fasting plasma glucose is known to decrease after the third month of pregnancy,^34^ a pattern reflected in our time analysis of CVD data, which showed a decrease in glucose over time in both the GDM and non-GDM groups (Supporting Information Table S4). Decreased plasma glucose between the first and third trimesters may have contributed to the observed decrease in ADP and uracil.

Two bile acids, taurocholate and glycocholate, increased over time. Higher bile acids have been associated with elevated insulin resistance across pregnancy,^35^ so the increase observed in this study may be a reflection of the natural insulin resistance that increases between the first and third trimesters. As reviewed by McIlvride et al., bile acid homeostasis is regulated by the farnesoid X receptor (FXR), a hepatic and intestinal nuclear receptor that, when activated by elevated bile acids, reduces bile acid synthesis in a negative feedback loop. FXR has been shown to have reduced activity during pregnancy, and insulin resistance has been observed in FXR knockout in mice. As such, the reduced FXR activity in pregnancy may be contributing to both elevated bile acids and insulin resistance, with elevated insulin resistance allowing for more glucose availability for the fetus.^36^

Sorbitol increased in both the GDM and the non-GDM groups over time. Periods of hyperglycemia have been shown to upregulate the sorbitol–aldose reductase pathway, a pathway that reduces excess glucose to sorbitol, which is then oxidized to fructose.^37^ Additionally, a study of early pregnancy found sorbitol to be more abundant in GDM cases compared to healthy controls.^38^ Because plasma glucose decreases in late pregnancy,^34^ and because CVD risk data and dietary data found blood glucose and carbohydrate intake to decrease over time, the elevation in sorbitol is counterintuitive. However, a study investigating fetal and maternal plasma concentrations of polyols in the third trimester found increased umbilical venous concentrations of sorbitol. This potentially indicates placental production of sorbitol or transport of sorbitol from maternal circulation,^39^ though further research is required to understand the mechanism behind this elevation.

Strengths of this study include the use of matched samples from a RCT, thus reducing bias by utilizing a double-blind method. The inclusion of food record data makes it less likely that the metabolomic differences observed between groups were due to diet. The observed similarity in dietary data between groups, in combination with covariate adjustment for age, BMI, ethnicity, and treatment group, improves the ability to detect differences that are specifically due to GDM. Additionally, samples were obtained from two sites on opposite coasts of the United States, thus allowing for results that were a more accurate representation of a larger population.

This study also had some weaknesses, most notably the reduced capability to generalize to the GDM population at large due to the small sample size. The decision for sample size was based on the number of participants from the Healthy Beginnings trial that both gave consent for their samples to be used for ancillary analyses and had sufficient remaining plasma samples from both a first trimester and third trimester blood collection. An additional analysis of plasma collected at diagnosis of GDM in the second trimester would have provided more insight into GDM-associated metabolomics alterations across pregnancy, as the third trimester plasma collection occurred several weeks after GDM diagnosis. The timing of this collection made it difficult to determine if third trimester metabolite alterations were due to GDM management methods, such as the observed decrease in estimated carbohydrate intake over time in the GDM group. Additionally, the 24 h dietary recall is subject to limitations, as it is self-administered and therefore may not be an accurate reflection of actual dietary intake. Another limit was utilization of the specific targeted metabolites of the metabolomics assays, as an untargeted metabolomics approach may have resulted in more metabolite differences between groups. The PLS-DA analysis was incapable of clearly differentiating groups. However, univariate analyses confirmed significant differences for individual metabolites. The ability to fully interpret the study findings was further limited by the lack of quantitative data. Additional analysis of the metabolites observed to be altered in our semi-quantitative metabolomics assays via quantitative, validated, clinical assays would provide valuable information that could provide improved insight into the observed changes. Future research with a larger sample size, second trimester metabolomics analysis, an untargeted metabolomics approach, and quantitative assays would allow for a more complete assessment of multi-trimester metabolites associated with GDM.

## Conclusions

Metabolomic profiling in GDM compared to non-GDM pregnancies across the first and third trimesters found a decrease in plasma medium-chain acylcarnitines in the GDM group over time, as well as an increase in purine degradation metabolites in the GDM group. The metabolite dysregulation observed in GDM-affected pregnancies may provide insight into mechanisms of GDM pathogenesis or serve as markers of the consequences of GDM development. Further research into the mechanisms behind these metabolite alterations is necessary to gain an improved understanding of GDM development. Additionally, GDM studies investigating changes in medium-chain acylcarnitines over time are limited, highlighting the need for further investigation into the role of medium-chain acylcarnitines in GDM.

## Data Availability

All raw data and metadata will be available through the NIH Metabolomics Workbench under Study ID: ST001948.^40^

## Supporting Information Available

PLS-DA showing the distribution of GDM vs Non-GDM samples in the first trimester (Figure S1); PLS-DA showing the distribution of GDM vs Non-GDM samples in the third trimester (Figure S2); group-by-time dietary intake comparisons and test of interactions (Table S1); group-by-time physical activity comparisons and test of interaction (Table S2); group-by-time metabolomics comparisons and test of interaction (Table S3); and time effect for cardiometabolic risk marker data (Table S4) (PDF)

## Abbreviations

ADP: adenosine diphosphate
BMI: body mass index
CrOT: carnitine octanoyltransferases
FAO: fatty acid oxidation
FXR: farnesoid X receptor
GDM: gestational diabetes
IMP: inosine monophosphate
NGT: normal glycemic tolerance
OGTT: oral glucose tolerance test
PLS-DA: partial least squares discriminant analysis
RCT: randomized controlled trial
T1D: type 1 diabetes
T2D: type 2 diabetes
