# Supplementary material for: Gestational Diabetes Is Characterized by Decreased Medium-Chain Acylcarnitines and Elevated Purine Degradation Metabolites across Pregnancy: A Case–Control Time-Course Analysis
Source: J Proteome Res. 2023 May 2;22(6):1603–13. doi: 10.1021/acs.jproteome.2c00430 (PMC10243101; doi:10.1021/acs.jproteome.2c00430)
Supplement: Supplementary file 1 — pr2c00430_si_001.pdf [file pr2c00430_si_001.pdf]

Gestational diabetes is characterized by decreased medium-chain acylcarnitines and elevated purine degradation metabolites across pregnancy: A case-control time-course analysis

Hannah Heath[1‡], Rodrigo Rosario[1‡], Lauren E. McMichael[1], Catherine M. Johnson[1], Rob Fanter[2], Noemi Alarcon[4,5], Adilene Quintana-Diaz[7,8], Kari Pilolla[1,5], Andrew Schaffner[5,6], Elissa Jelalian[7], Rena R. Wing[7], Alex Brito[8,9], Suzanne Phelan[4,5], Michael R. La Frano[1,3,5,#][1]Department of Food Science and Nutrition, California Polytechnic State University, San Luis Obispo, CA, 93407

[2]College of Agriculture, Food and Environmental Sciences, California Polytechnic State University, San Luis Obispo, CA, 93407

[3]Cal Poly Metabolomics Service Center, California Polytechnic State University, San Luis Obispo, CA, 93407

[4]Department of Kinesiology and Public Health, California Polytechnic State University, San Luis Obispo, CA, 93407

[5]Center for Health Research, California Polytechnic State University, San Luis Obispo, CA, 93407

[6]Department of Statistics, California Polytechnic State University, San Luis Obispo, CA, 93407

[7]Department of Psychiatry and Human Behavior, Warren Alpert Medical School at Brown University, Providence, RI, 02903, USA

[8]Laboratory of Pharmacokinetics and Metabolomic Analysis. Institute of Translational Medicine and Biotechnology. I.M. Sechenov First Moscow State Medical University, 119991, Moscow, Russia

[9]World-Class Research Center "Digital biodesign and personalized healthcare", I.M. Sechenov First Moscow State Medical University, 119991, Moscow, Russia

[‡]Hannah Heath and Rodrigo Rosario contributed equally to warrant co-first authorship.

# Corresponding Author: Michael R. La Frano, PhD, RD, Department of Food Science and Nutrition, Center for Health Research, California Polytechnic State University, San Luis Obispo, 1 Grand Ave, San Luis Obispo, CA 93407. Email: mlafrano@calpoly.edu

Supporting Information Table of Contents

Figure S1. PLS-DA showing distribution of GDM vs Non-GDM samples in the 1st trimester

Figure S2. PLS-DA showing distribution of GDM vs Non-GDM samples in the 3rd trimester

Table S1. Group by time dietary intake comparisons and test of interactions

Table S2. Group by time physical activity comparisons and test of interaction

Table S3. Group by time metabolomics comparisons and test of interaction

Table S4. Time effect for cardiometabolic risk marker data.

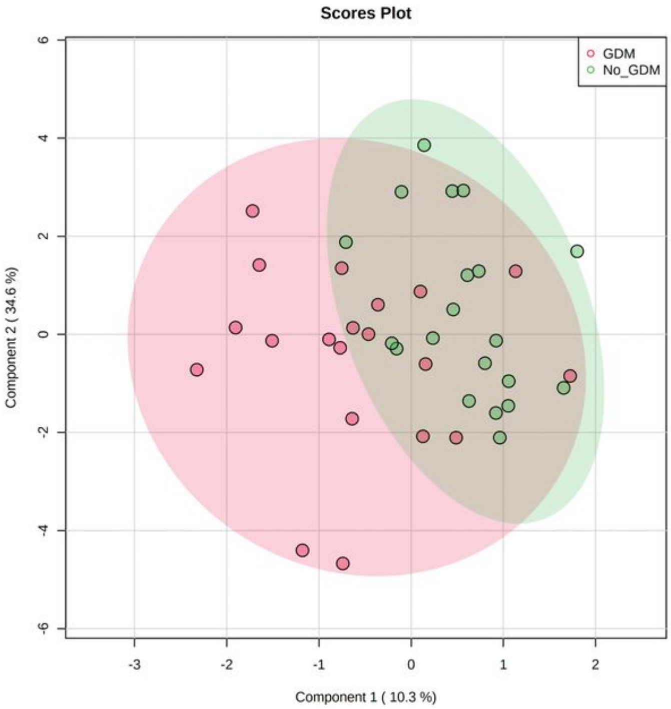

Supplementary Figure S1. Scores plot from an orthogonal partial least squares discriminatory analysis showing the distribution of the GDM vs. Non-GDM samples in the 1st trimester.

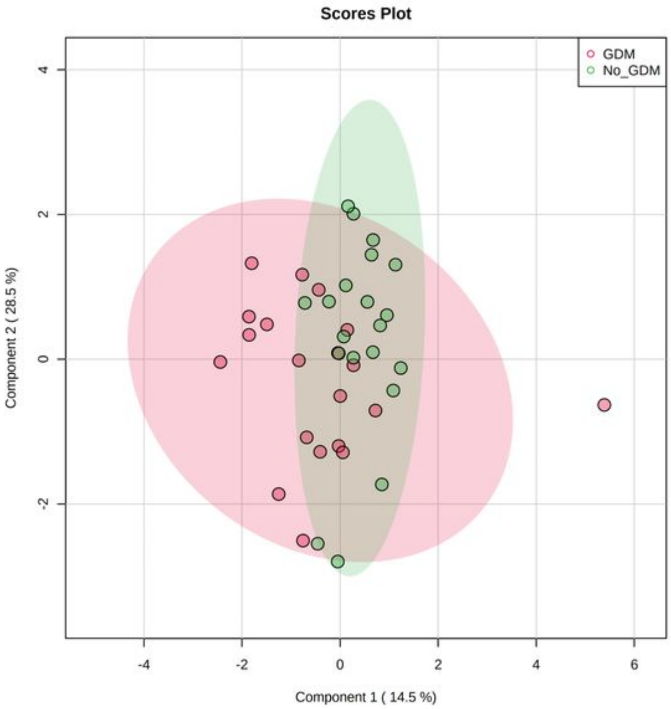

Supplementary Figure S2. Scores plot from an orthogonal partial least squares discriminatory analysis showing the distribution of the GDM vs. Non-GDM samples in the 3rd trimester.

Supplemental Table S1. Group by time dietary intake comparisons and test of interaction

Metabolite	Trimester	*Non-GDM*		*GDM*		P-value
		**Mean**	**SD**	**Mean**	**SD**	
Carbohydrates, %Kcal	1st	51.4%	10.8%	49.5%	7.7%	0.17
	3rd	51.8%	7.9%	46.8%	8.4%	
Fat, %Kcal	1st	18.0%	5.0%	17.5%	4.0%	0.27
	3rd	17.1%	3.1%	17.9%	4.2%	
Protein, %Kcal	1st	32.5%	8.7%	34.1%	5.0%	0.42
	3rd	32.5%	6.3%	36.5%	7.2%	
Calories, Kcal	1st	1693.0	526.5	1963.1	453.2	0.23
	3rd	1632.2	360.8	1652.6	557.8	
Total fat, g	1st	60.8	26.3	74.3	21.8	0.78
	3rd	60.0	19.3	69.3	26.8	
Saturated fat, g	1st	21.2	11.4	23.4	6.8	0.57
	3rd	19.7	6.9	22.9	10.7	
Monounsaturated fat, g	1st	22.0	9.4	27.4	9.0	0.99
	3rd	21.7	8.5	24.8	10.1	
Carbohydrates, g	1st	221.7	97.0	243.7	63.9	0.04
	3rd	209.8	47.0	187.9	69.6	
Cholesterol, mg	1st	212.4	84.0	315.3	212.3	0.92
	3rd	249.5	145.5	318.9	208.6	
Sodium, g	1st	2894.2	904.7	3520.2	843.8	0.09
	3rd	2712.5	786.9	2779.5	998.8	
Sugar, g	1st	109.0	73.5	105.9	42.1	0.15
	3rd	97.9	30.0	78.9	35.5	
Fiber, g	1st	16.2	8.6	16.6	5.7	0.56
	3rd	13.8	7.6	13.1	6.0	
Protein, g	1st	72.9	20.7	85.2	28.4	0.42
	3rd	69.0	18.8	74.1	28.7	
Potassium, mg	1st	2325.2	747.8	2421.6	801.3	0.36
	3rd	2257.9	708.3	2083.5	743.6	
Iron, mg	1st	68.2	73.3	54.1	47.2	0.59

	3rd	59.8	36.3	40.9	9.9	
Vitamin D, µg	1st	16.4	6.0	16.0	7.6	0.45
	3rd	19.8	20.3	17.4	10.1	
Vitamin A, µg RAE	1st	367.6	231.5	423.2	258.4	0.95
	3rd	495.1	305.3	549.8	279.3	
Folate, µg	1st	1394.8	488.6	1321.1	539.4	0.65
	3rd	1265.3	577.4	1175.6	300.9	
B12, µg	1st	16.9	8.2	38.1	113.8	0.96
	3rd	13.5	5.7	11.6	3.2	
Alpha-carotene, µg	1st	106.7	164.3	559.1	746.6	0.03
	3rd	311.1	482.7	231.8	233.4	

*Data presented represents Daily Total Nutrients from Foods and Supplements (TNS)

Abbreviation: Non-GDM, non-gestational diabetes mellitus; GDM, gestational diabetes mellitus; SD, standard deviation; RAE, retinol activity equivalent

Supplemental Table S2. Group by time physical activity comparisons and test of interaction

Physical activity	Trimesters	*Non-GDM*		*GDM*		P-Value
		Mean	SD	Mean	SD	
Accelerometry Data						
Accelerometer worn	1st	1415.6[1]	13.6	1407.5	21.4	0.80
	3rd	3335.4	3710.0	1950.8	2166.3	
Light activity	1st	1305.3	60.8	1316.2	58.8	0.89
	3rd	1313.7	61.7	1323.3	35.5	
Moderate activity	1st	132.4	60.4	121.6	58.3	0.88
	3rd	122.2	59.3	115.6	35.4	
Vigorous activity	1st	2.1	1.9	2.2	2.4	0.34
	3rd	2.0	2.3	1.1	0.7	
Sedentary activity	1st	995.0	108.2	1023.2	83.8	0.66
	3rd	993.2	114.5	1013.4	66.7	
Non-sedentary activity	1st	445.0	108.2	416.8	83.8	0.49
	3rd	444.7	113.2	426.6	66.7	
Locomotion	1st	13.9	8.6	15.3	9.9	0.09
	3rd	13.5	7.2	10.8	6.5	
Non-locomotion	1st	1426.1	8.6	1424.7	9.9	0.04
	3rd	1424.3	11.0	1429.2	6.5	
Physical Activity Questionnaire Data						
Walking pace (mph)	1st	2-2.9	< 2	2-2.9	< 2	0.04
	3rd	< 2	< 2	2-2.9	< 2	
Walking (mins/week)	1st	60	15-19	60	15-19	0.21
	3rd	60	5-19	20-59	5-19	
Lap swimming (mins/week)	1st	1-4	1-4	1-4	1-4	0.81
	3rd	1-4	1-4	1-4	1-4	
Weight training with arm weights (mins/week)	1st	1-4	5-19	1-4	5-19	0.94
	3rd	1-4	1-4	1-4	1-4	
Weight training with leg weights (mins/week)	1st	1-4	5-19	1-4	5-19	0.95
	3rd	1-4	1-4	1-4	1-4	

[1]Minutes per day, all accelerometry data values

Supplementary Table S3. Group by time metabolomics comparisons and test of interaction

Metabolite	Trimester	*Non-GDM*		*GDM*		P-value
		Mean	SD	Mean	SD	
Decanoylcarnitine	1st	174549	81051	239942	151319	0.01
	3rd	180616	82273	143849	67053	
Lauroylcarnitine	1st	119163	38510	159880	110840	0.03
	3rd	102701	50987	77695	33143	
Octanoylcarnitine	1st	42204	21904	52096	28164	0.03
	3rd	41891	20885	32361	15556	
Decenoylcarnitine	1st	79466	35919	103554	36903	0.04
	3rd	71171	22962	70889	29575	
Tyrosine	1st	492421	194478	444108	226898	0.06
	3rd	383702	160205	446001	164070	
Methionine	1st	708381	410028	595135	405184	0.07
	3rd	586219	423932	721664	357903	
Uridine	1st	524582	163699	639439	206953	0.07
	3rd	514968	176974	497763	163821	
Uracil	1st	296204	114790	327570	113495	0.07
	3rd	264386	92718	229216	76769	
Propionylcarnitine	1st	726775	497522	650585	549549	0.08
	3rd	455354	410046	580343	512110	
Inosine	1st	14391	13518	17543	18327	0.09
	3rd	13594	11370	30001	25404	
4-pyridoxate	1st	1087466	2825859	438137	359764	0.09
	3rd	426333	519788	420781	245593	
Dodecenoylcarnitine	1st	190292	86236	215326	110045	0.10
	3rd	173245	97347	129276	54191	
5-hydroxyindoleacetic acid	1st	24554	16534	22753	15954	0.11
	3rd	20331	13543	28319	13370	
Carnitine	1st	6433094	4124787	5083689	3977002	0.11
	3rd	4719469	3475468	4779454	2647771	
3-	1st	37198	27907	28545	31336	0.13

Methylglutarylcarnitine	3rd	35586	44284	46810	40236	
Alpha-hydroxybutyrate	1st	1589962	574001	2058695	918528	0.13
	3rd	1383531	384638	1397649	578636	
Glucose	1st	256260	167327	207590	180643	0.13
	3rd	180992	141276	189415	116174	
Cis-trans-hydroxyproline	1st	8384600	4431797	7704689	4846285	0.15
	3rd	5993997	4083029	7389998	3366074	
IMP	1st	23602	17572	27986	24521	0.16
	3rd	15404	7455	28049	15586	
Kynurenine	1st	83363	106808	113262	118287	0.16
	3rd	99060	114873	72794	114955	
Glutarylcarnitine	1st	32033	24507	23072	17261	0.16
	3rd	28568	30172	28649	16751	
Glucosamine	1st	377545	118326	369949	112197	0.18
	3rd	345618	110780	412165	108756	
Indoleacetate	1st	122631	68221	118372	88733	0.18
	3rd	133260	88281	161594	76442	
Octenoylcarnitine	1st	134165	77043	146509	97367	0.18
	3rd	108140	85423	129776	53700	
Isoleucine	1st	7492334	4008204	6904409	4502873	0.18
	3rd	5468326	3762992	6519810	3070108	
Citrate	1st	965787	877632	1093274	722604	0.18
	3rd	1295873	1033320	991988	604244	
Glycocholate	1st	83360	66174	101830	114122	0.20
	3rd	150150	151037	261660	252458	
2-hydroxyglutarate	1st	144338	98825	162881	210784	0.23
	3rd	148752	113335	184858	130591	
Creatine	1st	3200831	2060433	2862159	1922392	0.23
	3rd	2761612	2034652	3344291	2152052	
Tetradecenoylcarnitine	1st	61020	31626	74558	41323	0.24
	3rd	59348	33565	53946	31774	
Betaine	1st	18260575	9883197	16487934	11202201	0.26
	3rd	16144936	8366629	17301309	8021767	

Leucine	1st	9401339	5178580	8777407	5661391	0.27
	3rd	6959083	4679751	8416009	4321368	
Beta-hydroxybutyrate	1st	500393	322750	854237	916013	0.27
	3rd	807736	712411	794588	668381	
3-OH-hexanoylcarnitine	1st	42263	31671	35994	27785	0.27
	3rd	40158	39957	43177	25697	
Symmetrical dimethylarginine	1st	843113	417806	775701	463321	0.27
	3rd	841572	445093	934292	443897	
SUM_Taurodeoxycholate_taurochenodeoxycholate	1st	18258	16893	17923	16183	0.27
	3rd	12033	6081	13811	8975	
Myristoylcarnitine	1st	24269	11401	29882	15684	0.28
	3rd	25992	12467	24287	8463	
Glutamate	1st	368489	211566	329875	210439	0.29
	3rd	345242	191280	393232	213350	
Hypoxanthine	1st	189362	101725	259896	145073	0.30
	3rd	200933	87017	228910	134701	
Taurocholate	1st	19672	14684	26156	17595	0.31
	3rd	54390	41559	135638	238470	
Adipate	1st	513164	233111	511914	231067	0.32
	3rd	557960	271779	449613	224973	
Dihydroxyacetone phosphate	1st	14913	12264	16029	14086	0.32
	3rd	20234	26140	20092	14818	
Tryptophan	1st	4288227	2095725	4160065	2269341	0.33
	3rd	3482199	1747000	3974827	1522034	
Malate	1st	34242	9349	35598	13013	0.33
	3rd	32906	14212	28824	11548	
Choline	1st	7063721	4531486	6362351	4829649	0.34
	3rd	7620408	5317999	7898716	4650643	
Adenosine monophosphate	1st	6760	3436	22878	68285	0.34
	3rd	5958	2716	39371	131561	
Valine	1st	7065020	3052454	6401779	3559237	0.35
	3rd	5788255	3145324	5853283	2465725	
Aspartic acid	1st	430514	179067	383260	137251	0.35
	3rd	426382	179783	444116	175756	

2-aminoadipate	1st	1583092	2702071	1297574	2563564	0.36
	3rd	3284339	7549838	2856478	5003806	
Taurine	1st	323472	145929	517303	807884	0.36
	3rd	260393	100031	455672	899345	
SUM_hexose_diphosphates	1st	8359	6256	7159	6273	0.36
	3rd	6060	4368	7066	5147	
Phosphotyrosine	1st	121103	42839	121397	38693	0.38
	3rd	130272	45600	141875	39192	
SM C22:0	1st	1241450000	151038327	1202350000	252433060	0.38
	3rd	1193000000	235393064	1262100000	283564139	
Allantoin	1st	21377	9893	25609	18912	0.39
	3rd	25996	13045	25457	31660	
Histidine	1st	14044552	7960311	12097429	6934939	0.40
	3rd	13807628	8875630	13729818	6584104	
Alpha-ketoglutarate	1st	1175190	787216	1211210	962946	0.41
	3rd	1669267	1960267	1076343	312721	
N-Monomethyl L-arginine	1st	78027	42007	76921	49230	0.41
	3rd	76292	33865	86799	41649	
Indole-3-propionate	1st	186759	147187	192682	172475	0.42
	3rd	171381	138618	172129	112734	
Hypoxanthine	1st	51048	36782	66789	57744	0.43
	3rd	35725	15704	60907	37787	
Threonine	1st	346556	217080	282642	185456	0.47
	3rd	420224	228370	401627	187751	
SM C24:1	1st	780050000	131981847	782021760	225219559	0.47
	3rd	673800000	223673540	704380487	188612816	
3-OH-palmitoleoylcarnitine	1st	16822	10067	22468	14537	0.48
	3rd	13413	6800	17492	14995	
Sorbitol	1st	454844	381746	443932	378183	0.51
	3rd	594539	757973	517314	193581	
SM C16:1	1st	262000000	33370330	258895871	50402682	0.51
	3rd	2602500	43155624	27552630	5815544	

		00		3	0	
Proline	1st	8205467	4144980	7882675	4733359	0.52
	3rd	8323434	4472349	8744980	3838835	
SM C15:0	1st	59814999	14466278	57677075	18202635	0.52
	3rd	54258312	19583890	55826817	15574726	
Oleylcarnitine	1st	463765	174329	565019	363312	0.52
	3rd	379520	185128	529589	429423	
Dimethyl L-arginine	1st	1248016	561143	1128162	583356	0.53
	3rd	1371566	591659	1416039	701577	
Xanthine	1st	66432	24740	78961	26471	0.54
	3rd	64345	28947	68252	26032	
PC C34:4	1st	100140714	35042413	102758631	31073893	0.55
	3rd	94329098	33009181	90673858	30515966	
Nicotinamide	1st	39383	17291	109704	292820	0.56
	3rd	34443	10104	130105	422740	
Acetylcholine	1st	451059	322543	397119	329059	0.56
	3rd	376649	337332	362741	235250	
Pantothenate	1st	35318	33984	42520	37124	0.57
	3rd	39487	25268	54293	61754	
Phosphoenolpyruvate	1st	122904	22218	125219	26731	0.58
	3rd	117544	26728	122704	22848	
Glutamine	1st	16428742	7069636	14806512	6577581	0.59
	3rd	15593633	7035637	15535019	5516528	
Urate	1st	147522	64729	152717	82917	0.59
	3rd	170463	83351	185263	93925	
Serine	1st	494163	291234	439032	264921	0.60
	3rd	441285	265377	409909	214237	
Palmitoleoylcarnitine	1st	95934	54696	115129	62419	0.60
	3rd	83711	43495	90824	48265	
Adenine	1st	117616	44220	116694	43215	0.61
	3rd	120971	64005	123103	39431	
Triiodothyronine	1st	2442	1281	2333	1102	0.62

	3rd	2058	1125	2317	1434	
Serotonin	1st	128912	64514	131990	86699	0.62
	3rd	137424	79948	157191	93425	
SUM_fumarate_malate	1st	946436	1076231	988566	1217992	0.63
	3rd	1338280	1547017	947722	1136004	
Dimethylglycine	1st	780445	405410	711879	415641	0.63
	3rd	809488	492428	775143	380172	
Asparagine	1st	513996	253778	488414	282655	0.67
	3rd	523292	346228	508501	219263	
LPC C16:0	1st	798800000	168116689	826000000	130068808	0.67
	3rd	770700000	191113111	778750000	162329384	
Cytidine	1st	23965	19113	23548	12017	0.67
	3rd	22738	13612	31611	22718	
Phenylalanine	1st	5278387	2013628	5401063	2180562	0.68
	3rd	5380406	2414427	5769676	1698462	
Cytidine monophosphate	1st	11624	4404	17854	31883	0.69
	3rd	7916	3401	15866	34503	
Alanine	1st	1834195	979081	1841657	1278760	0.71
	3rd	1855480	1205314	1827508	1063170	
LPC C20:4	1st	35677929	13870554	38098481	7898445	0.72
	3rd	29964657	11112917	34013116	10639903	
SUM_fructose_glucose_galactose	1st	22274651	4862221	23269364	5722131	0.72
	3rd	22232846	7126455	22166243	5973671	
Alpha-glycerophosphate	1st	100043	44588	101192	45204	0.72
	3rd	84859	41584	107022	97941	
Creatinine	1st	1350967	653253	1401099	826850	0.73
	3rd	1416796	833562	1481028	631280	
Inosine monophosphate	1st	32932	16368	49694	79270	0.73
	3rd	21359	8272	62388	163409	
Adenosine diphosphate (ADP)	1st	34974	13217	36974	12209	0.74
	3rd	20406	9289	22222	8199	
SUM_Malonyl/3-	1st	42866	31852	67058	54235	0.74

OH-butyrlcarnitine	3rd	61723	54348	76708	52093	
N-carbamoyl beta-alanine	1st	245520	130428	279108	204073	0.75
	3rd	229544	102989	242420	129111	
Inositol	1st	25275	14312	31026	25428	0.75
	3rd	37281	25802	43744	23431	
Pyruvate	1st	17415	6943	17429	7472	0.75
	3rd	17383	7864	17920	8902	
LPE C22:6	1st	361600000	122967433	391300000	91093994	0.76
	3rd	323200000	136187410	342200000	113030317	
HIppurate	1st	341946	253010	308669	193028	0.77
	3rd	385436	269868	374878	187295	
Lysine	1st	14645466	6656084	13796108	6449648	0.78
	3rd	13904233	6436865	13897580	5089121	
Alpha-glycerophosphocholine	1st	379378	318004	324515	203097	0.78
	3rd	309679	306120	448540	517886	
SM C14:0	1st	224600000	34259689	217044222	45294153	0.79
	3rd	214650000	47897121	213303776	50819614	
SM C22:1	1st	856200000	133345850	861500000	135015594	0.79
	3rd	858150000	168542756	849500000	181186586	
SM C24:0	1st	330150000	85237300	336595915	140303187	0.81
	3rd	289747751	136029159	298024272	98539158	
Arginine	1st	5416998	2348746	5024368	1959669	0.82
	3rd	4817465	2151254	4870530	2490307	
Inosine	1st	7972	2586	13272	13486	0.82
	3rd	10495	4850	14668	10641	
SM C18:0	1st	331400000	68017335	332100000	91848216	0.83
	3rd	350400000	87979902	336639067	87213861	
Glycine	1st	18404	9405	17230	14407	0.83
	3rd	19755	14814	15325	8805	

Kynurenic acid	1st	17765	9301	17705	9741	0.84
	3rd	17096	7929	18533	11241	
Indoxylsulfate	1st	3809	2795	3917	2993	0.84
	3rd	3530	2712	3839	2708	
Acetylcarnitine	1st	11435585	5064398	11273382	6169041	0.85
	3rd	9710870	5216248	9182298	3575290	
Palmitoylcarnitine	1st	395516	129575	527613	226574	0.86
	3rd	333923	136424	492060	530447	
PC C34:3	1st	293750000	61121858	292800000	69965104	0.87
	3rd	297050000	70334894	299017848	76086292	
Linoleylcarnitine	1st	223197	97106	284132	168016	0.88
	3rd	175940	83017	253539	213126	
Niacinamide	1st	151631	70835	400923	1071408	0.89
	3rd	116857	35488	500348	1645003	
Alanine 2	1st	1843117	918905	1890837	1334833	0.91
	3rd	1922100	1324600	1761472	1028146	
Ornithine	1st	596080	297355	656660	511032	0.91
	3rd	529723	267127	579579	314999	
Thyroxine	1st	91987	31668	95233	31396	0.92
	3rd	81565	26849	83527	32850	
SUM_Butyrl/Isobutyrylcarnitine	1st	731915	389522	772139	408182	0.92
	3rd	624932	327090	624685	365507	
SM C20:0	1st	1516500000	145395106	1541050000	207873817	0.92
	3rd	1640000000	206576098	1676400000	311995682	
Uridine-5-monophosphate	1st	26341	14282	37733	77415	0.93
	3rd	17777	7321	27610	55035	
LPC C18:2	1st	648600000	146307679	677000000	128025491	0.94
	3rd	661150000	175339153	690250000	196830645	
Citrulline	1st	501411	248411	490827	306754	0.94
	3rd	491807	244657	485775	243184	
Lactate	1st	5344659	2172930	6259263	3225192	0.95
	3rd	5554053	2054832	6021360	1847480	

SUM_glycodeoxycholate_glycochenodeoxycholate	1st	336047	289743	567728	626470	0.95
	3rd	396821	304885	624787	801350	
PC C36:3	1st	850600000	148249221	875300000	138467591	0.95
	3rd	840550000	141518262	873650000	177212116	
PC C38:6	1st	357450000	97842771	356000000	64623607	0.97
	3rd	326500000	78966015	328800000	74771652	
Xanthosine	1st	92022	36366	103699	86912	0.98
	3rd	86677	39318	89260	39950	
3-hydroxyanthranilicacid	1st	186031	79774	195085	99554	0.99
	3rd	191782	73854	232190	227519	

Mean and SD data are in peak area

Abbreviations: Non-GDM, non-gestational diabetes mellitus; GDM, gestational diabetes mellitus; SD, standard deviation

Metabolites ranked by p-values.

Supplementary Table S4. Time effect for cardiometabolic risk marker data

CVD Risk Factor	*1st Tri Non-GDM (n=20)* Mean	*3rd Tri Non-GDM (n=20)* Mean	*1st Tri GDM (n=20)* Mean	*3rd Tri GDM (n=20)* Mean	P-value
Triglycerides (mg/dL)	133 ± 63.9[1]	244.6 ± 85.5	148.5 ± 61.5	240.7 ± 92.8	2.9E-11
Total Cholesterol (mg/dL)	181.0 ± 32.4	234.5 ± 43.0	176.8 ± 35.9	211.6 ± 38.4	3.1E-10
Glucose (mg/dL)	90.3 ± 8.3	85.6 ± 7.0	91.9 ± 8.8	85.3 ± 8.3	5.8E-07
C-Peptide (ng/mL)	2.5 ± 1.5	3.6 ± 1.1	2.9 ± 1.0	3.8 ± 1.6	1.9E-05
LDL Cholesterol ¥	89.1 ± 26.9	121.8 ± 38.3	89.8 ± 32.9	106.5 ± 35.1	2.3E-05
HDL Cholesterol †	65.3 ± 14.7	65.3 ± 18.2	57.3 ± 12.4	58.8 ± 17.5	0.14
Leptin(ug/ L)	54.2 ± 15.2	61.2 ± 12.8	52.6 ± 14.2	63.8 ± 26.5	0.37
HOMA-IR	4.6 ± 4.5	4.5 ± 2.5	4.9 ± 2.8	4.8 ± 2.2	0.39
Insulin (uU/mL)	18.9 ± 16.0	20.3 ± 10.6	21.0 ± 10.9	22.5 ± 9.3	0.91

Abbreviations: Non-GDM, non-gestational diabetes mellitus; GDM, gestational diabetes mellitus

¥Friedewald LDL Cholesterol

†HDL Cholesterol, Direct

[1]Mean ± standard deviation (all such values)
